# Microstructure and Mechanical Properties of Narrow Gap Laser-Arc Hybrid Welded 40 mm Thick Mild Steel

**DOI:** 10.3390/ma10020106

**Published:** 2017-01-26

**Authors:** Chen Zhang, Geng Li, Ming Gao, XiaoYan Zeng

**Affiliations:** 1 School of Mechanical & Electrical Engineering, Wuhan Institute of Technology, Wuhan 430073, China; c_zhang@hust.edu.cn; 2 Wuhan National Laboratory for Optoelectronics (WNLO), Huazhong University of Science and Technology, Wuhan 430074, China; ligeng_mark@foxmail.com (G.L.); xyzeng@mail.hust.edu.cn (X.Z.)

**Keywords:** narrow gap, hybrid welding, microstructure, mechanical properties

## Abstract

Both laser-arc hybrid welding and narrow gap welding have potential for the fabrication of thick sections, but their combination has been seldom studied. In this research, 40 mm thick mild steel was welded by narrow gap laser-arc hybrid welding. A weld with smooth layer transition, free of visible defects, was obtained by nine passes at a 6 mm width narrow gap. The lower part of the weld has the lowest mechanical properties because of the lowest amount of acicular ferrite, but its ultimate tensile strength and impact absorbing energy is still 49% and 60% higher than those of base metal, respectively. The microhardness deviation of all filler layers along weld thickness direction is no more than 15 HV_0.2_, indicating that no temper softening appeared during multiple heat cycles. The results provide an alternative technique for improving the efficiency and quality of welding thick sections.

## 1. Introduction

Recently, thick sections of low carbon/low alloy steel were widely applied in modern industries, such as shipbuilding, bridge manufacturing, pressure vessel fabrication, etc. Narrow gap (NG) welding is one of the preferred techniques to reduce cross sectional area, improve welding efficiency, enhance the performance, and decrease residual stress of the welded thick sections. 

NG arc welding (NGAW) is one of the most widely used techniques for joining thick sections. In order to keep the stability of arc burning, the groove width is at the range of 10–18 mm or even wider. As the width of the arc was about 6–8 mm, a lack of fusion easily occurs in NGAW. Gao and Wang [1,2] studied the effect of wire rotation on weld formation, especially lack of fusion in NGAW. Yao, Chen and Yamane [3,4,5] demonstrated that the process stability and weld morphology of NGAW could be improved by controlling the twisting and moving of the wire. Fan [6] found that twin-wire technique could improve arc stability, avoiding lack of fusion by adjusting wire feeding rate and wire distance. In conclusion, besides some achievements, the challenges of low efficiency, lack of fusion and poor mechanical performances still exist in NGAW.

In the last decade, NG laser welding (NGLW) has developed rapidly. Zhang [7,8] used 6 kW disc-laser to fill 50 mm thick SUS316L stainless steel by eight passes, and 10 kW fiber laser to fill the same thickness by only four passes. The test showed that these NGLW welds have a lower residual stress than tungsten inert gas (TIG) welded joint. Tsukamoto [9] joined 60 mm thick SA-516 low carbon steel by multi pass NGLW with 10 kW fiber laser. He found that 60 mm thick plate could be fulfilled by 10 passes, but lack of fusion also occurred due to the low heat input. Elmesalamy [10] used 1 kW single mode fiber laser to weld 20 mm thick 316L stainless steel in heat conduction mode at a welding groove of 1.5 mm by 18 passes. Wang Baiping [11] welded 35 mm thick Q345 mild steel by NGLW but found some lack of fusions within the weld. Since the laser beam spot size is less than 0.5 mm, the requirements of groove preparation and beam-wire position are quite strict for NGLW, which does not benefit the joining of thick sections [12,13,14].

Laser-arc hybrid welding (LAHW) has been one of the techniques with the most potential for fusion welding in decades [15], offering deep penetration, stable processing, high welding speed and great adaptability under severe conditions. Materials with thickness of 15–28 mm could be penetrated at one pass by LAHW with a laser power higher than 10 kW [16,17,18]. Because of these advantages, LAHW started to be employed in modern industries. For example, Meyer Werft shipyard [19] used this technique to weld decks with a thickness of 15 mm, with an efficiency that was three times higher than conventional submerged-arc welding. Jokinen [20] made a comparison between LAHW and laser welding of 20 mm thick AISI304L stainless steel, which demonstrated that LAHW has a higher efficiency. Aubert [21] welded 25 mm thick duplex stainless steel with 14 passes using 10 kW Nd:YAG LAHW, whose efficiency was 10 times higher than arc welding. These advantages showed that LAHW had potential to improve the fabrication efficiency and joint performances of welded thick sections when incorporated into NG welding.

However, no attention has been paid to narrow gap laser-arc hybrid welding (NGHW) with a high power fiber laser. Therefore, our group studied NGHW of mild steel in detail, and achieved a high quality welding joint. This article mainly focuses on microstructure and mechanical properties achieved by NGHW of 40 mm thick mild steel.

## 2. Experimental

An IPG fiber laser (IPG Photonics Corporation, Oxford, MA, USA) with a wavelength of 1070 nm and maximum output power of 6 kW was employed. Its beam parameter product is 6.9 mm × mrad. The laser beam was transferred to the laser head through an optical fiber with a 200 nm core diameter, and then collimated by a 150 mm lens and focused by a 250 mm lens. The focus spot diameter of laser beam was 0.4 mm. A Fronius TPS4000 gas metal arc welding (GMAW) machine with a maximum electric current of 400 A was used. The arc used in this study was in pulse mode. Both laser head and arc torch were fixed to the flange of a Fanuc M-710iC six axes robot with repeat accuracy of ±0.05 mm.

**Figure 1:**
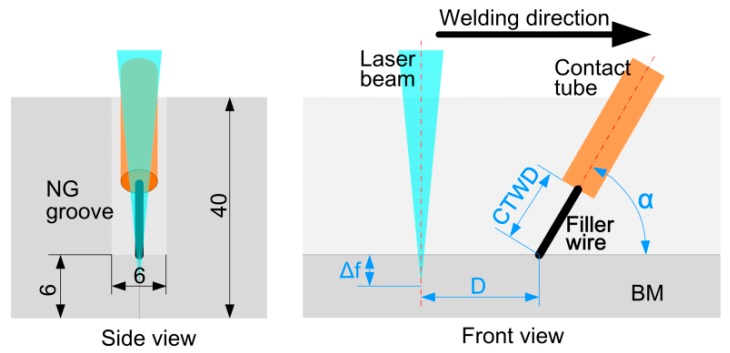
Schematic diagram of groove dimension (side view) and experimental setup (front view), where *D* is distance between beam center and wire tip, Δ*f* is focal point position, α is angle of arc torch to workpiece surface, CTWD is distance between contact tube and workpiece.

The base material (BM) was 40 mm thick hot rolled and annealed Q235 mild steel. The filler wire was ER70S-6 with a 1.0 mm diameter. Shielding gas of arc torch was Ar + 18%CO_2_ at a flow rate of 20 L·min^−1^. Those material compositions and mechanical properties are shown in Table 1. Before welding, the NG groove was machined as shown in Figure 1, and the oxidized film on the groove surface was removed by stainless steel brush and cleared by acetone.

The experiment set-up is shown in Figure 1. The heat source arrangements and the welding parameters of different passes are shown in Table 2. The welding was carried out in arc leading mode. After welding, metallurgical samples were prepared by GB/T 13298-91 [22], which were etched by nitric acid alcohol solution (2% by volume) with etching time of 10 s. Weld microstructure was observed by Zeiss optical microscope (OM). The contents of acicular ferrite in different zones were calculated by software of Image-Pro Plus. Impact fractures were examined by FEI Sirion 200 scanning electron microscope (SEM) at an accelerating voltage of 15–20 kV. Chemical compositions of weld local areas were examined by energy dispersive X-ray spectroscopy (EDS) at 20 kV.

**Figure 2:**
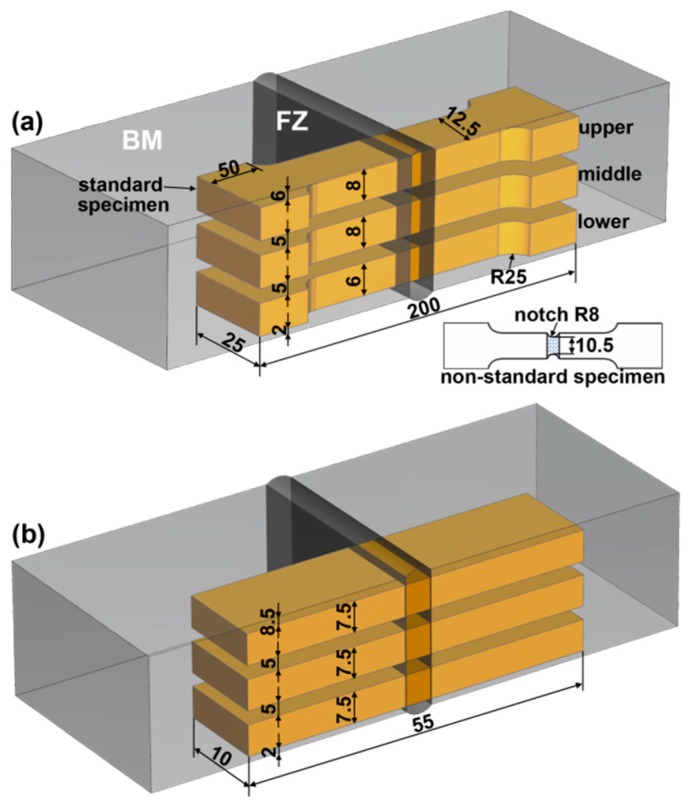
Cutting location and dimension size of the specimens, (**a**) ultimate tensile strength (UTS); (**b**) impact absorbing energy (IAE).

**Figure 3:**
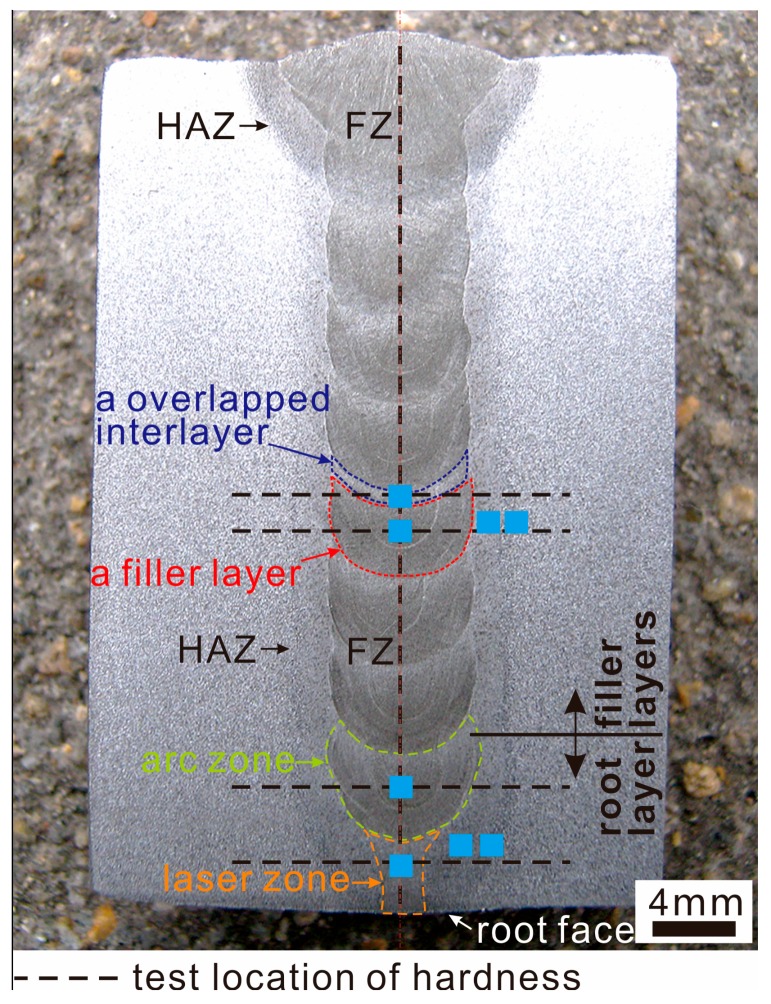
Cross section of 40 mm thick joint created with narrow gap laser-arc hybrid welding (NGHW). The areas for examining microstructure zone are marked by blue cubes and the locations for testing microhardness are marked by dash lines.

Ultimate tensile strength (UTS) and impact absorbing energy (IAE) of the weld were measured in terms of the standards of ISO 4136:2012 [23] and ISO 9016:2012 [24], respectively. Considering the weld was composed of multi-layers, both tensile and impact specimens were collected from upper, middle, and lower parts. The cutting location and the dimension of the specimens are shown in Figure 2. Because all standard tensile specimens cracked at the BM, a non-standard specimen with notch at the fusion zone (FZ) was designed to evaluate the UTS of the FZ of different areas. The results are the average of three specimens. Vicker microhardness was evaluated across the surface of metallurgical samples using a 1.96 N load for 20 s. The loading locations of the microhardness test are shown by the dash line in Figure 3.

## 3. Results and Discussion

### 3.1. Microstructure

As shown in Figure 3, an accepted weld is obtained by nine passes. It is without visible defects and has smooth transition between the passes. According to the shape and microstructure features, the weld is characterized by root layer, filler layer, and overlapped interlayer between filler layers. The locations which microstructures are taken from are shown in Figure 3.

**Figure 4:**
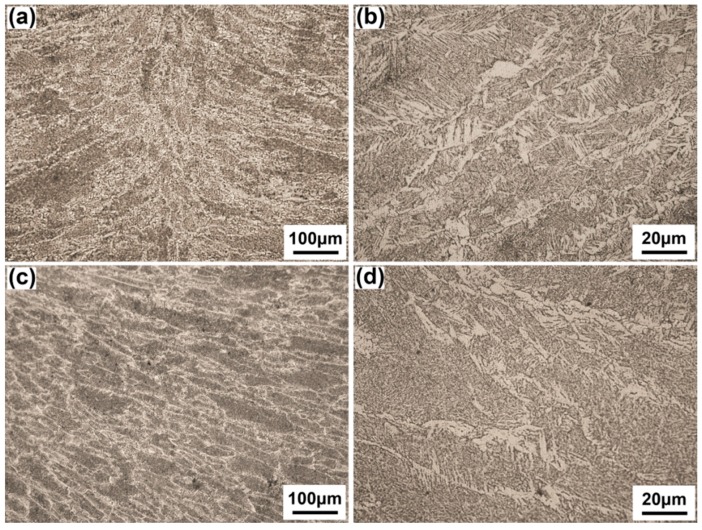
Microstructures of root layer, (**a**) laser zone; (**b**) amplified picture of (**a**); (**c**) arc zone; (**d**) amplified picture of (**c**).

The root layer is divided into arc zone and laser zone according to the features of joints created by LAHW [25]. The width of the arc zone is wider than that of laser zone. In Figure 4a, the laser zone is composed of columnar dendrites growing from fusion line to weld center because the narrower molten pool and the faster cooling rate promotes the quick growth of columnar dendrites [26]. In Figure 4c, the arc zone is still composed of dendrites but with coarser grain size because the accumulation of arc heat causes a bigger heat input at this area. Besides, the content of acicular ferrite of the arc zone (60.3%) is higher than that of the laser zone (48.9%).

**Figure 5:**
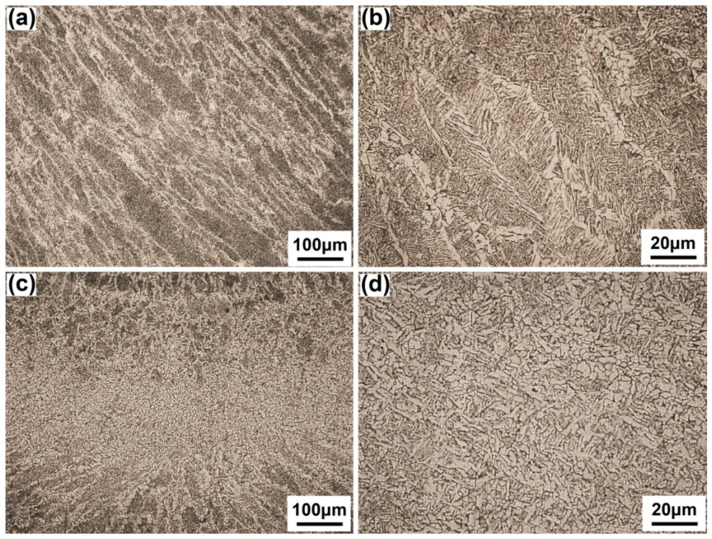
Microstructure of filler layer and overlapped interlayer, (**a**) filler layer; (**b**) amplified picture of (**a**); (**c**) overlapped interlayer; (**d**) amplified picture of (**c**).

In Figure 5a,b, the filler layer is composed of acicular ferrite, side plate ferrite and a small amount of proeutectoid ferrite, which is similar to the arc zone of the root layer. The average grain size and content of acicular ferrite are 60.15 μm and 52.4%, respectively. In Figure 5c,d, the overlapped interlayer is a second heated microstructure at the top part of the last layer. It is a tempered microstructure composed of fine ferrite grain, and has the average grain size of 5.83 μm with a thickness of approximately 410 μm.

**Figure 6:**
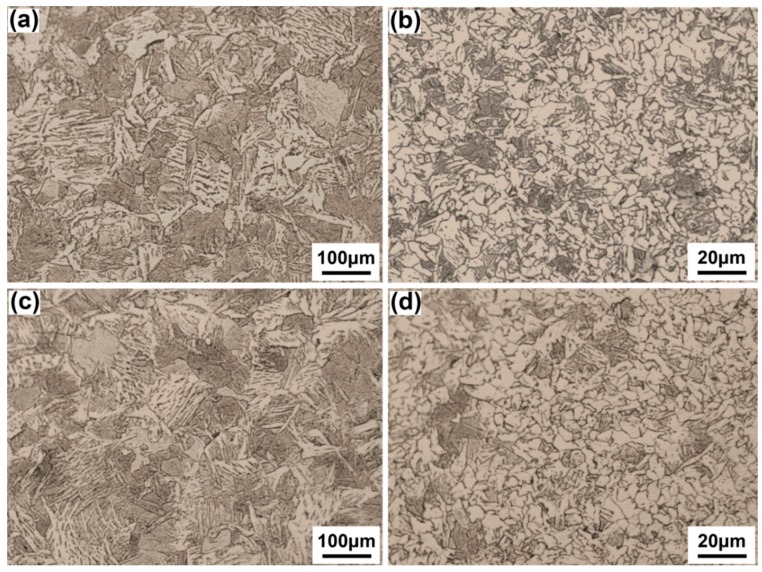
Microstructure of heat affected zones (HAZ), (**a**) coarse grain zone of root layer; (**b**) fine grain zone of root layer; (**c**) coarse grain zone of filler layer; (**d**) fine grain zone of filler layer.

In Figure 6, the heat affected zones (HAZ) of both the filler layer and the root layer are composed of a coarse grain region and a fine grain region. The coarse grain region is composed of coarse widmannstatten structure because it is overheated at the temperature range between 1100 °C and solid phase line during welding. The fine grain region consists of fine pearlite and ferrite that result from recrystallization at the austenite transformation temperature range between 1000 °C and the critical transformation temperature line (Ac3). The HAZ of the filler layer has smaller grain size than that of the root layer because of lower heat input.

**Figure 7:**
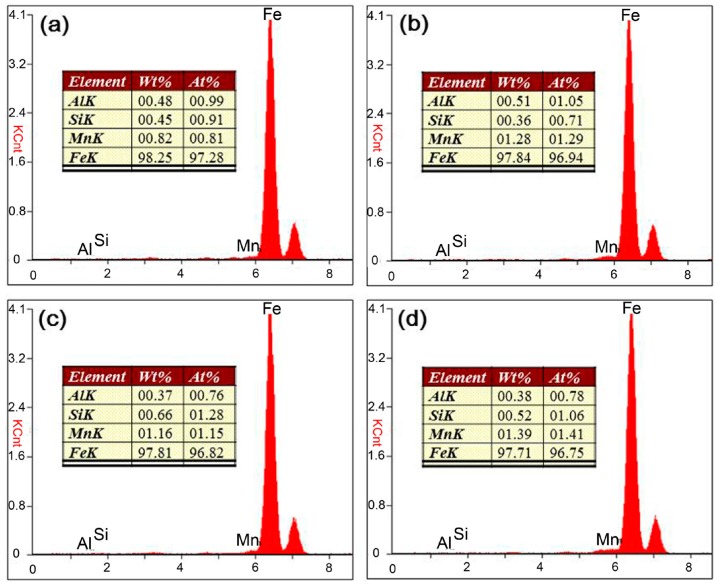
Energy dispersive X-ray spectroscopy (EDS) component analysis of different zones within the weld, (**a**) laser zone of root layer; (**b**) arc zone of root layer; (**c**) overlapped interlayer; (**d**) filler layer.

In Figure 7, the Mn content of the laser zone of the root layer is 0.82%, obviously lower than other zones. These zones have the characteristics of arc welding, which creates stronger melt pool convection by arc pressure and droplet impact force. On the other hand, these zones were mainly made of the filler wire. All these factors favor the incorporation of the Mn from filler wire, and cause higher Mn content in the filler layer, overlapped interlayer, and arc zone of the root layer compared to the laser zone of the root layer. Besides, the Mn content in the arc zone of the root layer is lower than in the filler layer because Mn content is diluted by molten BM in the laser zone.

### 3.2. Microhardness

**Figure 8:**
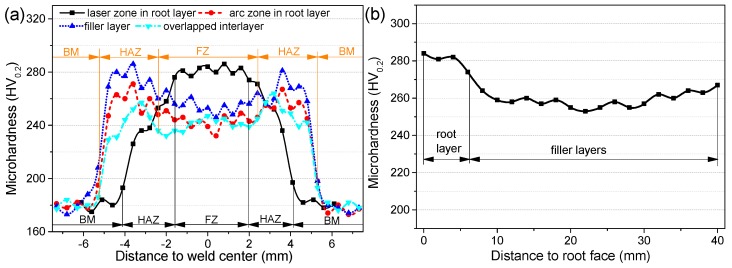
Microhardness profiles across weld surface, (**a**) profiles along horizon direction of different layers; (**b**) profile along thickness direction of weld center.

In Figure 8a, three findings are found. Firstly, the microhardness of both the FZ and the HAZ are higher than that of the BM. Secondly, the FZ microhardness of the laser zone of the root layer is the highest, while that of the overlapped interlayer is the lowest. The average FZ microhardness of the laser zone of the root layer is 20–40 HV_0.2_ higher than that of other layers. Thirdly, the laser zone of the root layer has a smooth transition because of the highest FZ microhardness, while an obvious hardening zone corresponding to the HAZ appears within other layers because their FZ microhardness is lower than that of the HAZ. Obviously, the laser zone of the root layer has the features of laser weld with the quickest cooling rate and the narrowest HAZ within the weld [27], as well as the highest microhardness because of the finest grains. On the other hand, since the FZs of other layers have coarser grains and mainly consist of ferrite, their microhardness is lower than that of corresponding HAZs consisting of widmannstatten structure, which causes the HAZ to be a hardening zone.

In Figure 8b, the microhardness profile along thickness direction of weld center shows that except for the root layer, full filler layers have uniform microhardness due to the same welding parameters. As a whole, the microhardness difference is no more than 15 HV_0.2_, indicating that no temper softening appears within the weld along thickness direction.

### 3.3. Tensile Strength and Impact Toughness

Since all the tensile specimens fracture at BM, the UTS of FZ are featured by the notch specimen. Table 3 shows that the UTS of whole weld is about 712 MPa, and the UTS and the IAE gradually decrease from the upper specimen to the lower specimen. The UTS of FZ, the IAE of FZ, and the IAE of HAZ of the upper specimens increase 0.3%, 1.2% and 1.5% higher than those of the middle specimens, and 3.9%, 8.3% and 14.7% higher than those of the lower specimens, respectively. Even the lower specimens with the lowest performance have UTS of FZ, IAE of FZ, and IAE of HAZ that are 49%, 60%, and 32% higher than BM, respectively. The results indicate that the mechanical properties of the joint created by NGHW are excellent. 

The mechanical properties correspond well with the microstructure. The lower specimens of the laser zone of the root layer have the lowest content of acicular ferrite because of the quick cooling rate and lower Mn content. As the acicular ferrite is the main structure to improve the toughness of the low carbon steel joint [28], the lower specimen has the weakest mechanical properties. The mechanical properties of the middle specimen are a bit weaker than those of the lower specimen because of the partial tempering under multiple heating cycles and lower content of acicular ferrite.

**Figure 9:**
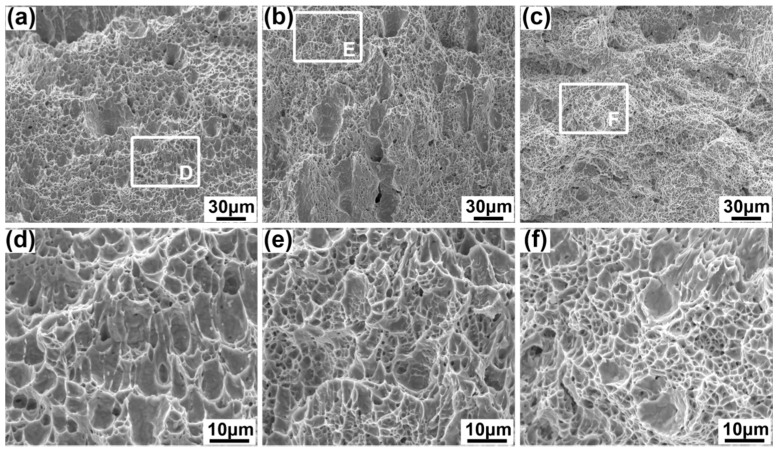
Fracture morphologies of the fusion zone (FZ) impact specimen at different parts, (**a**) the upper specimen; (**b**) middle specimen; (**c**) lower specimen; (**d**) details of area D; (**e**) details of area E; (**f**) details of area F.

In Figure 9, all impact fracture surfaces are characterized by the dimples, but the average size of the dimples decease from the upper to the lower, which are 5.86 μm, 5.47 μm and 4.73 μm for the upper, middle, and lower specimens. The larger the IAE, the smaller the dimple size.

## 4. Conclusions

(1) An accepted weld of 40 mm thick mild steel was obtained by narrow gap laser-arc hybrid welding with nine passes at a 6 mm width narrow gap. The weld was with smooth layer transition and free of visible defects.
(2) The weld could be characterized by some typical layers with different microstructure, which are the laser zone of the root layer, the arc zone of the root layer, the filler layer, and an overlapped interlayer between filler layers. The laser zone of the root layer had the lowest content of acicular ferrite within the fusion zone (48.9%), while the arc zone of root layer had the highest content of acicular ferrite (60.3%).
(3) At the laser zone of the root layer, the fusion zone had the highest microhardness, 20–40 HV_0.2_ higher microhardness than the fusion zone of other layers. The microhardness transition was smooth from the fusion zone to the base metal along the horizon direction of the laser zone, while an obvious hardening zone appeared in other layers because the microhardness of their fusion zone was lower than their heat affected zone. The microhardness deviation of all filler layers along weld thickness direction was no more than 15 HV_0.2_, indicating that no temper softening appeared during multiple heat cycles.
(4) The lower part of the weld had the lowest mechanical properties because of the lowest amount of acicular ferrite, but its ultimate tensile strength and impact absorbing energy were still 49% and 60% higher than those of base metal, respectively, indicating that the weld had a good performance.

## Figures and Tables

**Table 1:** Nominal chemical compositions and mechanical properties of the base material (BM) and filling wire (weight %—wt %), where YS is yield strength, ultimate tensile strength (UTS) is ultimate tensile strength, EL is total elongation, and impact absorbing energy (IAE) is impact absorbing energy.

Materials	Compositions (wt %)							Mechanical Properties			
	C	Si	Mn	P	S	Others	Fe	YS (MPa)	UTS (MPa)	EL (%)	IAE (J)
**BM**	0.12	0.15	0.44	0.015	0.008	≤0.3	Bal.	≥235	450	26	120
**Filling wire**	0.08	0.92	1.52	0.020	0.015	≤0.5	Bal.	≥420	550	30	150

**Table 2:** Welding parameters, where *P* is laser power, *I* is arc current, *U* is arc voltage, *V_f_* is wire feed rate, *PF* is pulse frequency of *I*, *T* is inter pass time, *v* is welding speed.

Parameters	Root Layer Welding	Filler Layer Welding
*D* (mm)	3	3
Δ*f* (mm)	−2	−2
*α* (°)	55–60	55–60
*CTWD* (mm)	10–12	10–12
*P* (W)	3000	800
*I* (A)	210	230
*U* (V)	24.1	24.9
*V_f_* (m·min^−1^)	7.2	7.7
*PF* (Hz)	177.4	188.7
*T* (s)	-	300
*v* (m·min^−1^)	0.4	0.4

**Table 3:** UTS and IAE of different parts within the weld. FZ: fusion zone

Location	UTS	IAE	
		FZ	HAZ
Whole weld	712.5 ± 15.0	-	-
Upper specimen	697.6 ± 14.6	208.3 ± 12.3	181.7 ± 10.7
Middle specimen	695.3 ± 14.6	205.7 ± 12.1	179.0 ± 10.6
Lower specimen	671.4 ± 14.1	192.3 ± 11.3	158.3 ± 9.3
